# Facilitators and barriers in obtaining informed consent for neonatal research: a scoping review

**DOI:** 10.1007/s00431-026-06865-y

**Published:** 2026-04-08

**Authors:** Kelly K. Storm, Veroni Stolk, Wes Onland, Sylvia A. Obermann-Borst, Anne Smits, Irwin K. M. Reiss, Anton H. van Kaam, Sinno H. P. Simons, G. Jeroen Hutten

**Affiliations:** 1 Department of Neonatal and Pediatric Intensive Care, Division of Neonatology, Erasmus University Medical Center – Sophia Children’s Hospital, Rotterdam, The Netherlands; 2 Department of Neonatology, Emma Children’s Hospital, Amsterdam UMC, Amsterdam, The Netherlands; 3 Amsterdam Reproduction & Development Research Institute, Amsterdam, The Netherlands; 4 Care4Neo, Neonatal Patient and Parent Advocacy Organization, Rotterdam, The Netherlands; 5 Neonatal Intensive Care Unit, University Hospitals Leuven, Leuven, Belgium; 6 Department of Development and Regeneration, KU Leuven, Leuven, Belgium

**Keywords:** Informed consent, Clinical research, Neonatology, Parental decision-making

## Abstract

Obtaining informed consent is crucial in clinical research. This process is particularly challenging in a Neonatal Intensive Care Unit. Parents of critically ill neonates already experience high emotional distress, and soliciting consent for research may exacerbate this burden. As evidence-based medicine is essential to improve neonatal care, clinical trials in neonates require adequate and non-selective enrollment. This study aims to find facilitators and barriers influencing the consent process in neonatal research. A scoping review was conducted using PubMed, Embase, Web of Science, Cochrane CENTRAL, CINAHL, and Google Scholar to identify peer-reviewed articles published in English, focusing on factors influencing parental decisions regarding their child’s enrollment in neonatal research. Identified factors were thematically categorized and labeled based on the level of evidence and direction of influence. From the 2108 retrieved articles, 20 were included for analysis. Common facilitators of research participation were perceived benefits for the child, altruistic motives to benefit or help other children, and the wish to contribute to knowledge and science. Notably, a higher severity of illness facilitated consent. Important barriers to participation were parental stress and fear, concerns about risks for the child, and the logistical burden of participation.

*Conclusion*: Obtaining consent in neonatal research is a complex process influenced by a nuanced balance between facilitators and barriers. The findings highlight the importance of providing adequate support to reduce the burden of participation. Through trust and clear communication, parents can feel empowered to make well-informed decisions, even in the most challenging situations.

**Table:** 

**What is Known:** • *Obtaining informed consent for research in the NICU is challenging due to the critical illness of the neonates, parental stress, and time constraints, often resulting in low and selective enrollment*.	
**What is New:** • *Parents are often motivated by perceived benefits, altruism, and the wish to contribute to knowledge and science, while parental fear, perceived risks, and burden of participation are barriers to research participatio*n.

**Supplementary Information:**

The online version contains supplementary material available at 10.1007/s00431-026-06865-y.

## Introduction

Obtaining informed consent is a crucial step in clinical research. It ensures that participants, or their legal representatives in pediatric or neonatal studies, fully understand the purpose, nature, and potential risks and benefits of the study before deciding to participate [1]. Recruiting children for research poses unique challenges, requiring careful consideration of the child’s best interest alongside parents’ rights and responsibilities in making decisions on behalf of their child. This is especially challenging in the Neonatal Intensive Care Unit (NICU), a high-stress environment where parents of critically ill neonates must deal with their child’s unstable condition, cope with emotional distress, and make complex medical decisions under time constraints [2, 3]. Soliciting consent in this context may increase parental distress even further [3].

Despite these challenges, robust clinical research in the NICU is crucial for improving neonatal outcomes. Many aspects of neonatal care, particularly pharmacological interventions, are supported by limited evidence, often extrapolated from pediatric or adult studies. This contributes to the frequent off-label medication use in this vulnerable group [4]. Participation in research is essential for generating knowledge and enabling evidence-based decisions. However, low and selective enrollment rates in neonatal studies pose an important obstacle, as they may lead to selection bias, limit generalizability, and prevent studies from reaching sufficient power or completing enrollment [5].

This scoping review summarizes existing literature on the factors that facilitate or hinder parental consent for neonatal research. By identifying key influences and knowledge gaps, it aims to improve the consent process and support adequate, non-selective enrollment in order to strengthen the evidence base in neonatal medicine.

## Materials and methods

### Protocol and registration

The Preferred Reporting Items for Systematic Reviews and Meta-Analyses extension for Scoping Reviews (PRISMA-ScR) guidelines were followed [6]. The protocol was registered in the Open Science Framework (10.17605/OSF.IO/U9KDT).

### Eligibility criteria

We included peer-reviewed articles written in English that investigated factors influencing informed consent for neonatal research in hospitalized neonates. Studies were excluded if parents were asked about hypothetical willingness to enroll their newborns, rather than reporting actual consent decisions.

### Search strategy

Six databases were searched: Medline, Embase, Web of Science Core Collection, Cochrane Central Register of Controlled Trials, CINAHL, and Google Scholar. Search terms combined concepts related to informed consent, research participation, and neonatal care (e.g., “consent”, “research participation”, “trial recruitment”, “neonatal intensive care”, “NICU”, “preterm”, “newborn”, “infant”). Full search strategies are available in Online Resource 1. Databases were searched from inception until April 22, 2025.

### Screening

After removing duplicates, two researchers (KS and VS) independently screened titles and abstracts, followed by full-text review. Disagreements were resolved by a third reviewer (GJH or SS).

### Data extraction

Two researchers (KS and VS) independently extracted data on publication year, setting, participants, methods, identified facilitators and barriers, and factors tested but not influencing consent. Discrepancies were resolved by discussion.

### Outcomes and synthesis

The primary outcome of this scoping review was factors influencing parental consent for neonatal research, identified through either qualitative or quantitative methods. Similar factors were first grouped together. We determined a priori that factors would be organized according to the level at which they resided. Within this overarching structure, categories were developed inductively through iterative sorting of the identified factors. This process resulted in five categories: (i) patient characteristics, (ii) parental characteristics and attitudes, (iii) relational factors, (iv) informed consent process, and (v) study characteristics. The initial categorization was performed by one researcher (KS) and cross-checked by a second researcher (VS).

Second, for each study, each identified factor was labeled based on the positive or negative direction of its influence and the level of evidence supporting its influence on research participation. The labels included *significant facilitator*, *facilitator*, *not of influence*, *barrier*, *or significant barrier*. In studies that compared parents providing or declining consent using statistical testing, each factor demonstrating a significant difference between the groups was labeled in both directions: as a *significant facilitator* when positively associated with providing consent and a *significant barrier* if it was negatively associated with providing consent. Factors showing no significant difference were labeled as *not of influence*. In descriptive studies without statistical testing, factors reported as influencing decisions were labeled simply as *facilitators* or *barriers*. Importantly, the distinction between “significant” facilitator/barrier and facilitator/barrier refers solely to the methodological basis of the evidence and does not imply greater substantive importance or impact.

### Quality appraisal

KS and GJH independently appraised study quality using either the Mixed Methods Appraisal Tool (MMAT) (7) or the Joanna Briggs Institute (JBI) Checklist for Systematic Reviews and Research Syntheses (8), depending on the study design. Conflicts were resolved by a third reviewer (SS).

## Results

### Study selection and characteristics

**Fig. 1:**
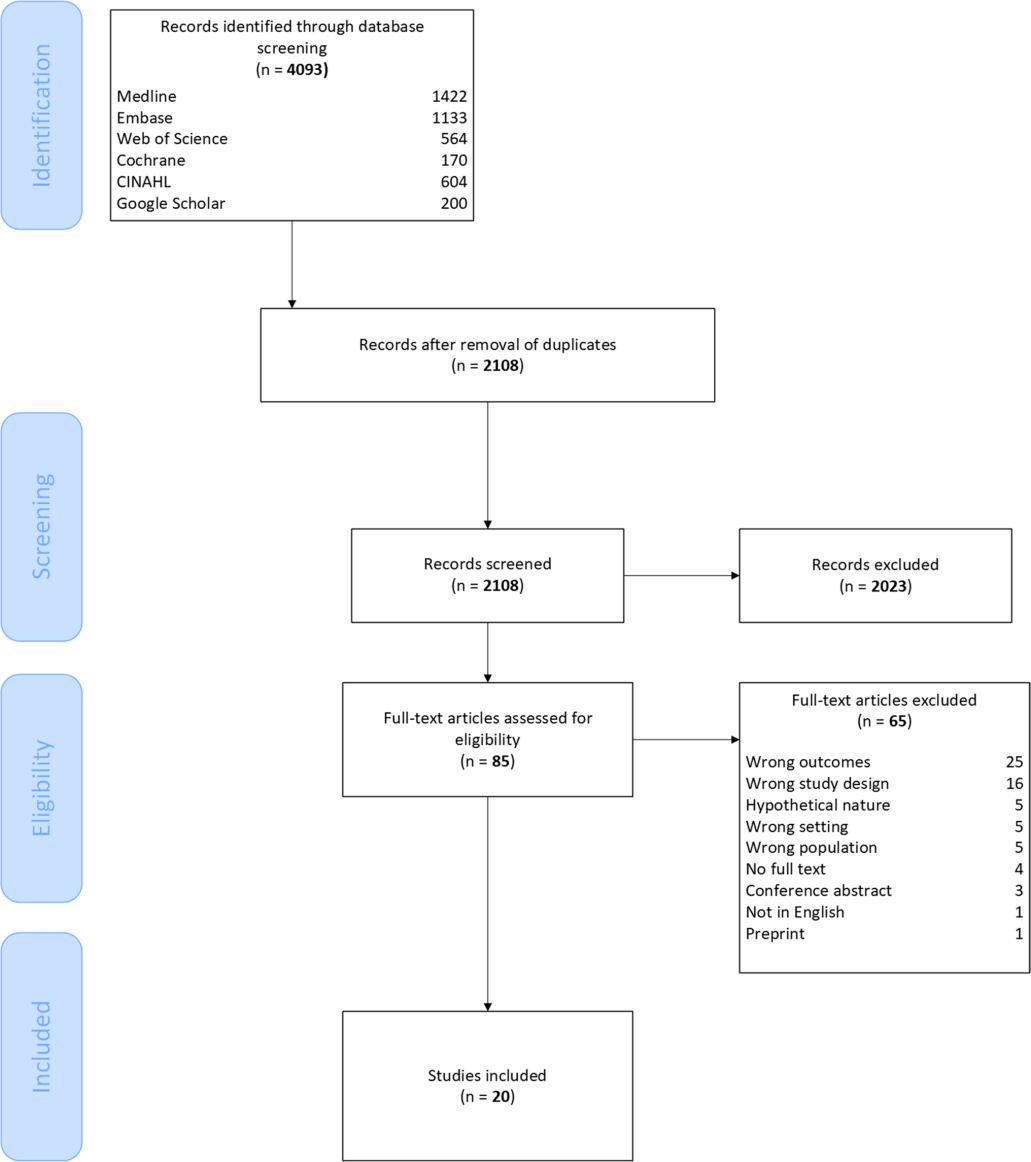
PRISMA flow diagram of the study selection process

A total of 2108 unique citations were identified by the search strategy for title and abstract screening. Of these, 85 were critically reviewed as full text, and 20 were retained for inclusion. The detailed study selection process is presented in the PRISMA flow diagram (Fig. 1), with the main reason for exclusion being the use of outcomes not aligned with our study aim. Interrater reliability assessment revealed moderate agreement for both abstract (96%, *κ* 0.59) and full-text screening (78%, *κ* 0.48).

The studies included in this review were conducted between 1997 and 2025, with sample sizes ranging from 15 to 281 parents, the majority of which originated from the USA. The studies included those who consented to research participation, declined research participation, or both consenters and decliners. A detailed overview of the study characteristics, including their quality appraisal, can be found in Table 1.

**Table 1:** Overview of the included studies

Author, year, country of origin	Study objective	Methodological approach	Design	Data source	Sample size	Participants	Contributed factors (+/−)	Quality appraisal*
**Ballard et al. 2004, USA **[7]	To determine the validity of informed consent obtained from parents of infants enrolled in the NEOPAIN study	Mixed-methods	Descriptive	Open-ended questionnaire	64	Consenters	5 (5/0)	2/5
**Bauer et al. 2021, Israel **[8]	To collect and explore information about parental attitudes and factors regarding consent for music intervention studies during their child’s NICU hospitalization	Quantitative	Cross-sectional	Questionnaire	203	Consenters and decliners	8 (6/2)	5/5
**Cakici et al. 2023, USA **[9]	To identify barriers to equitable enrollment of acutely ill newborns into a diagnostic genomic sequencing research study	Mixed-methods	Secondary analysis	Database	90	Decliners	7 (0/7)	5/5
**Cartwright et al. 2011, UK **[10]	To explore parents’ perceptions of their infants’ participation in randomized control trials (RCTs) and the implications of the RCT for their infant and themselves	Qualitative	Descriptive	Semi-structured interviews	16	Consenters	5 (5/0)	5/5
**Dahan et al. 2020, France **[11]	To explore how parents and physicians experience the informed consent interview in a tertiary NICU and identify factors influencing parents’ decisions, with a secondary focus on prenatal informed consent perspectives	Mixed-methods	Observational	Closed- and open-ended questionnaire	25	Consenters and decliners	11 (6/5)	5/5
**Hanvey et al. 2019, USA **[5]	To determine parental consent rates in neonatal drug trials and describe trial characteristics associated with higher rates	Quantitative	Review	Literature	38	Drug trials	4 (2/2)	6/11
**Hoehn et al. 2009, USA **[12]	To assess the impact of time on parental decision-making for research participation for neonates with congenital heart disease	Mixed-methods	Observational	Interview	37	Consenters and decliners	4 (1/3)	5/5
**Hulst et al. 2005, Netherlands **[13]	To examine whether parental authorization of involvement in a clinical study is influenced by the child's severity of illness at the time of the consent decision	Quantitative	Observational	Database	281	Consenters and decliners	4 (2/2)	4/5
**Israel et al. 2025, USA **[14]	To highlight the experiences and challenges encountered by a single study site in participant recruitment for an acute neonatal seizure treatment trial	Quantitative	Observational	Database	191	Consenters and decliners	2 (1/1)	5/5
**Mason et al. 2000, UK** **[**15]	To assess the validity of the informed consent process for parents in neonatal clinical trials across nine European countries and identify potential practical improvements	Qualitative	Descriptive	Semi-structured interviews	200	Consenters and decliners	9 (3/6)	4/5
**Nordheim et al. 2018, Norway **[16]	To examine how parents of very low birth weight infants experienced having their newborn infant enrolled in a randomized controlled intervention trial	Qualitative	Descriptive	Semi-structured interviews	15	Consenters	9 (7/2)	5/5
**O'Shea et al. 2018, Ireland **[17]	To determine parental and clinician views of the informed consent process in neonatal research	Mixed-methods	Descriptive	Closed- and open-ended questionnaire	43	Consenters and decliners	4 (2/2)	3/5
**Sawyer et al. 2017, UK **[18]	To explore women’s views and experiences of two alternative consent pathways to participate in the Cord Pilot Trial	Qualitative	Descriptive	Semi-structured interviews	23	Consenters	7 (7/0)	5/5
**Shah et al. 2018, USA **[19]	To explore the associations between the study personnel and timing of consent with parents’ decisional conflict and ultimately their decision to enroll	Mixed-methods	Observational	Closed- and open-ended questionnaire	163	Consenters and decliners	6 (4/2)	5/5
**Sloss et al. 2021, Australia **[20]	To evaluate the opinions of parents of newborns following their infant’s enrollment into a neonatal research study through the process of deferred consent	Mixed-methods	Observational	Structured interviews	100	Consenters and decliners	8 (5/3)	5/5
**van der Vaart et al. 2024, UK **[21]	To explore parent-reported motivation in deciding to participate in the Petal trial, and to understand parent-reported experiences related to trial participation	Quantitative	Descriptive	Survey	106	Consenters	4 (4/0)	5/5
**Weiss and Guttman et al. 2021, USA **[22]	To describe the parental experience of recruitment and assess differences between parents who participated and those who declined to enroll in a neonatal clinical trial	Quantitative	Observational	Survey and medical records	269	Consenters and decliners	20 (11/9)	5/5
**Weiss and Olszewski et al. 2021, USA **[23]	To assess differences in parental factors between parents who enrolled their infant and those who declined enrollment for a neonatal randomized clinical trial	Quantitative	Observational	Survey and medical records	269	Consenters and decliners	7 (4/3)	5/5
**Weiss et al. 2024, USA **[24]	To describe parents’ motivations for and against participation in neonatal research	Qualitative	Descriptive	Semi-structured interviews	44	Consenters and decliners	18 (6/12)	5/5
**Zupancic et al. 1997, Canada **[25]	To determine the extent to which parental decisions to enroll their infants in controlled clinical trials are influenced by risk and benefit considerations compared to other factors	Mixed-methods	Cross-sectional	Closed- and open-ended questionnaire	140	Consenters and decliners	10 (5/5)	5/5

*Quality appraisal according to the Mixed Methods Appraisal Tool (MMAT, out of 5) or the Joanna Briggs Institute checklist (JBI, out of 11)

### Factors of influence

**Fig. 2:**
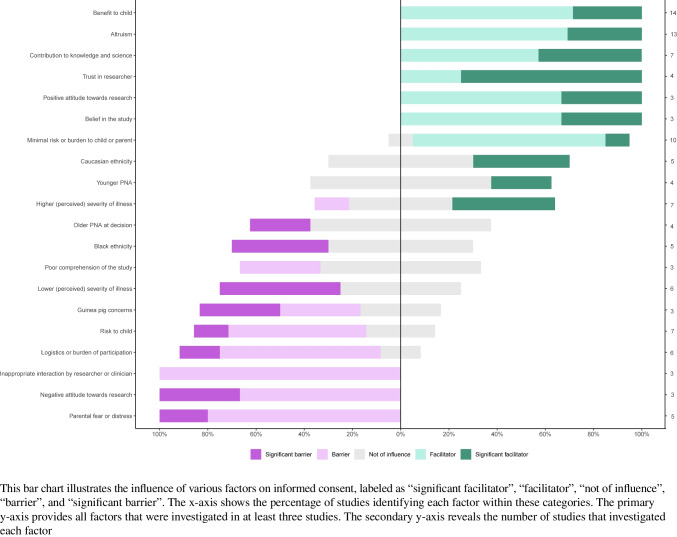
Facilitators and barriers to obtaining informed consent in neonatal research

Several key factors were identified as either facilitating or hindering the informed consent process. Figure 2 highlights those facilitators and barriers examined in three or more studies. A comprehensive overview of all identified factors that facilitated or hindered consent decisions can be found in Online Resources 2 and 3, respectively.

#### Patient characteristics

Among patient characteristics, the (perceived) severity of illness emerged as an important factor influencing parental consent. Six studies examined this relationship, of which three reported a significant association. Two studies found that parents were more likely to provide consent when their neonates were objectively more critically ill at birth, as indicated by a higher Clinical Risk Index for Babies (CRIB) score [8, 13]. In the third study, parental perception of a higher severity of illness, rather than objective measures, was associated with an increased likelihood of consent [23]. In the same study, however, severe encephalopathy did not distinguish between consenters and decliners. Conversely, when infants appeared less severely ill—either through objective CRIB scores or parental assessment—parents were less likely to consent [8, 13, 23]. In contrast, one study reported that parents declined participation specifically because they perceived their infant as “too sick or too small” [17].

Postnatal age (PNA) showed mixed associations. One study found that consenting parents tended to have significantly younger infants at the time of decision-making than parents that declined participation [8]. Other studies did not identify PNA as a factor influencing parental consent [13, 22, 23]. In preterm infants, a longer duration of NICU stay was linked to increased consent, whereas a shorter stay appeared to reduce the likelihood of consent [13].

#### Parental characteristics and attitudes

Several parental characteristics and attitudes emerged as influencing factors in the informed consent process for neonatal research. For the parental characteristics, two studies reported that parents identifying as White parents were significantly more likely to provide consent compared to parents identifying as Black [14, 23], whereas three studies found no association between parental ethnicity and consent decisions [8, 19, 22]. Additionally, one study identified an annual household income above $55.000 as a significant facilitating factor, and enrollment in Medicare, a public health insurance program in the USA, as significant barrier for consent [13].

Altruism was the most frequently reported facilitator among parental attitudes, with parents often motivated by the opportunity to benefit or help others [7, 8, 10, 11, 15–17, 19–22, 24, 25]. Closely related, several studies highlighted parents’ willingness to contribute to scientific knowledge as an important driver of consent [8, 11, 16, 19, 21, 22, 24]. Additional facilitators included belief in the value of the specific study [7, 18, 22], a generally positive attitude towards research [16, 18, 25], fear of regret declining participation [22], previous positive experiences with research [24], and a desire to “give back” through participation [24].

Among parental attitudes, parental fear and distress were the most commonly cited barriers to research participation, reported across five studies [9, 11, 15, 20, 22]. A recurring concern was the fear that their child would be treated as a “guinea pig” [22, 24], indicating unease about being among the first to experience a new intervention, although one study found this did not affect parental decisions [19]. A negative attitude towards research in general was reported as a barrier in three studies [15, 20, 25] and a lack of belief in the specific study in two [9, 20]. Parents experiencing decisional conflict, defined as “a state of uncertainty about a course of action” and assessed using the validated 16-item Decisional Conflict Scale [26, 27], were also more likely to decline participation [19, 22]. Other barriers included the fear of loss of control [22, 24], fear of discrimination [9, 24], preference for standard clinical care [24], privacy concerns [24], and a preference for consent to be obtained by a clinical team member [22].

#### Relational factors

Trust emerged as a critical theme in the parental consent process. Parental trust in the researcher or the individual obtaining consent was reported as a facilitator for providing consent [16, 19, 22, 23]. Trust in the medical team was also identified as a facilitator in one study [11], although another study found no influence on consent decisions [23]. Other relational aspects that supported informed consent included parents’ positive attitude towards the researcher [22], the perceived freedom to decide [25], the desire to be a “good” or cooperative patient on behalf of their child [11], and the willingness to help the researcher [20]. In contrast, inappropriate interactions with the researcher [11, 15, 24] and a lack of trust in the researcher [15, 19] were described as barriers. Lastly, feeling a sense of pressure or obligation was identified in one study as a reason for consent [11], but in another study as a barrier [25].

#### Informed consent process

In the informed consent process, the availability of sufficient time emerged as an important factor. Two studies reported that adequate time to decide facilitated participation [10, 12], whereas insufficient decision-making time was identified as a barrier [12, 22]. Beyond time, several studies highlighted the importance of how information was presented and processed. Parents who carefully read the consent form, had a positive first impression, and discussed their decision with others were significantly more likely to consent [22]. Furthermore, addressing parents’ concerns and questions [10], providing sufficient information to make a decision [16], and offering a clear explanation of the study [7] supported parental consent. Lastly, deferred consent was identified as a facilitator, as the baby “already received the intervention” [20].

Conversely, the absence of one parent during the consent process [11, 12] and too many proposals for other studies [11, 24] were identified as barriers in two studies. In addition, a poor comprehension of study information was highlighted in one out of three studies [11] and a complex consent process in one out of two studies [25]. Lastly, a negative first impression was a barrier [22].

#### Study characteristics

Perceived direct benefit of the study to the child was the most frequently reported facilitator of parental consent [7, 8, 10, 11, 15–22, 24, 25]. Closely following this, the belief that the study posed minimal risk or burden to the child was cited as a facilitating factor in nine out of ten studies [7, 10, 15, 16, 18, 21, 24, 25]. Other study-related characteristics that positively influenced consent decisions included parental belief in the research intervention [8, 18], the opportunity for the child to receive treatment [22], use of an active comparator instead of a placebo [5], parental benefits [18], and a shorter overall study duration, including treatment and follow-up, with rates significantly decreasing from 100% for studies lasting less than 24 h to 53% for those lasting more than 6 months [5].

In contrast, concerns about potential risks to the child, including side effects and safety, were cited as a barrier in five out of seven studies [15–17, 24, 25]. Logistical demands and burden of participation were also common obstacles, identified in five of six studies [12, 15, 16, 22, 24]. Other barriers included a perceived lack of direct benefit to the child [24, 25], concerns about the specific intervention (e.g., blood sampling, future use of data, genetic nature, or potential confidentiality risks) [9], a placebo-controlled design [5, 24], and longer study durations [5]. Randomization was highlighted as a concern in one out of two studies [24].

### Factors not of influence

Many other factors have been investigated for their role in informed consent but appeared not of influence. These factors are summarized in Online Resource 4.

## Discussion

This literature review revealed key facilitators and barriers in the parental informed consent process in neonatal research. Benefit to the child and altruism emerged as the most commonly identified facilitators to research participation, whereas parental fear and distress, along with the perceived risk to the child, emerged as the most reported barriers to research participation.

The identified facilitators were consistent with findings from previous reviews on pediatric research participation [28] and pediatric drug trials [29]. The facilitating effect of parents’ perception of direct benefit to their child reflects a natural prioritization of their child’s well-being—a pattern also observed in end-of-life decision-making contexts [30]. These expectations of direct benefits raise important ethical concerns, particularly when the anticipated benefits are uncertain or when parental understanding is influenced by incomplete or overly optimistic information. Such situations highlight the importance of clear, transparent, and balanced communication during the consent process to ensure that parents’ decisions are well-informed and ethically appropriate. Interestingly, beyond perceived benefits, the potential benefits of research participation may extend beyond the intervention itself. Some evidence suggests that neonatal trial participants, including those receiving a placebo, may experience outcomes at least as favorable as, if not better than, those of non-participants [31]. However, another study indicates that this apparent advantage may, at least in part, reflect selection bias rather than a true effect of trial participation [32]. Regardless, ensuring equitable access to research opportunities remains an ethical obligation.

The key barriers, including perceived risk to the child and parental fear and distress, were also consistent with previous reviews [28, 29] and emphasize the emotional burden parents face when making decisions on behalf of their newborn. Previous work by Weiss et al. emphasizes the importance of acknowledging the difficult circumstances under which parents are approached [33]. In addition, fear of potential harm may arise from uncertainties surrounding procedures or interventions. This underscores the value of not only clearly communicating risks but also providing reassurance through explaining safety measures.

An unexpected finding was that parents of infants with a higher (perceived) illness severity were more likely to consent to research participation. This contrasts with previous research suggesting that critically ill neonates may be less likely to be enrolled in clinical trials [32]. We had initially hypothesized that these parents, already under considerable stress due to their infant’s critical condition, would be more likely to refuse participation, as they might be overwhelmed with their infant’s care. In clinical practice, it is often considered burdensome to approach parents of severely ill infants, given their emotional distress and complex circumstances. One included study reported that parents declined participation because they felt their infant was “too sick or too small” [17]. Yet, three other studies identified a higher illness severity as a facilitator for consent [8, 13, 23]. Parents who perceive their infant as more severely ill may be more inclined to participate, viewing research as an opportunity to contribute to improved care or to “do everything possible” for their child. Increased contact with healthcare professionals in the context of critical illness may also foster trust and strengthen the therapeutic relationship, further encouraging consent. This apparent paradox highlights the importance of avoiding assumptions about parents’ willingness or ability to participate and, instead, ensuring that they are genuinely afforded the opportunity to make an informed decision themselves.

An important finding was that ethnic background emerged as a factor influencing parental consent in two studies, where White parents were significantly more likely to provide consent than Black parents. This concern is supported by Lyle et al. who demonstrated a persistent underrepresentation of Black, but also Asian, Hispanic, and Indigenous neonates in clinical trials [34]. Notably, fear of discrimination was also reported as a barrier to participation, referring to experiences of institutional racism, the Tuskegee experiment, and the case of Henrietta Lacks, where unethical research practices towards Black individuals led to mistrust in medical research. These observations point to the possibility that structural inequities and mistrust towards research or healthcare institutions may influence decision-making among minority groups. Ensuring that diverse populations are adequately represented is essential, both to promote equal opportunities and to generate evidence that is generalizable. Future studies should explicitly examine the factors underlying ethnic differences in consent decisions, such as discrimination, cultural perspectives, and trust, in order to identify strategies that foster inclusivity.

While our study provides valuable insights into the factors influencing the informed consent process for neonatal research, several limitations should be acknowledged. First, the limited number of included studies, often with small sample sizes, may reduce the robustness of individual findings and restrict their generalizability. Additionally, nearly half of the studies included in our review originated from the USA, which may influence the applicability of our findings to other healthcare systems and sociocultural settings, though many identified factors are likely relevant across different contexts. Lastly, the heterogeneity of study designs and contexts, including interventions as diverse as music therapy, drug trials, and genomic sequencing research, complicates the synthesis of results and limits the ability to draw overarching conclusions applicable across all neonatal research settings. Further in-depth analyses of already performed clinical trials, their characteristics and consent rates might further improve our understanding of obtaining parental consent.

### Implications for practice and future research

This review provides a structured synthesis of facilitators and barriers influencing parental consent in neonatal research, offering direction for future strategies aimed at improving recruitment and ensuring representativeness. Future strategies should focus on strengthening facilitators that were consistently identified across studies. Researchers should clearly communicate the potential benefits for the participating infant when applicable, as well as the broader contribution of research to knowledge necessary for improving outcomes of future patients. Fostering trust and relationship building [35] are important elements for research participation. To mitigate barriers, researchers should provide clear, tailored, and comprehensible information to reduce parental fear and uncertainty, address logistical burdens, and explain measures taken to reduce risk.

Several initiatives have already attempted to optimize the consent process, including educational interventions such as the Better Research Interactions for Every Family (BRIEF) and the Training tRial recruiters, An educational INtervention (TRAIN) [36, 37]. However, a systematic evaluation of interventions designed to improve consent is still lacking and would be valuable to assess effectiveness and identify best practices.

One critical area that remains underexplored is the researcher’s contribution to the informed consent process. One notable gap in the literature is the role of the researcher’s own perceptions in the consent decision of parents. Equipoise—the balance of perceived risks and benefits—may influence how research is framed and communicated to parents, potentially affecting their willingness to consent. Understanding how the researchers’ views impact the consent process could provide valuable insights into optimizing communication strategies.

## Conclusion

Obtaining consent in neonatal research is a complex process influenced by a nuanced balance between facilitators and barriers. While parents are often motivated by perceived benefits for their child, altruistic intentions, and belief in the importance of medical research, their decision-making can be hindered by emotional distress, concerns about risks, and logistical challenges. The findings highlight the importance of providing adequate support to reduce the burden of participation. Establishing trust and maintaining clear dialogue can enable parents, even under highly stressful conditions, to make informed and autonomous decisions.

## Supplementary Information

Below is the link to the electronic supplementary material.

Supplementary file1 (PDF 330 KB)

## Data Availability

No datasets were generated or analysed during the current study.

## Abbreviations

BRIEF: Better Research Interactions for Every Family
CRIB: Clinical Risk Index for Babies
GA: Gestational age
JBI: Joanna Briggs Institute
MMAT: Mixed Methods Appraisal Tool
NICU: Neonatal Intensive Care Unit
PNA: Postnatal age
PRISMA-ScR: Preferred Reporting Items for Systematic Reviews and Meta-Analyses extension for Scoping Reviews
TRAIN: Training tRial recruiters, An educational INtervention
