# Supplementary material for: Facilitators and barriers in obtaining informed consent for neonatal research: a scoping review
Source: Eur J Pediatr. 2026 Apr 8;185(5):244. doi: 10.1007/s00431-026-06865-y (PMC13061776; doi:10.1007/s00431-026-06865-y)
Supplement: Supplementary file 1 — Supplementary file1 (PDF 330 KB) [file 431_2026_6865_MOESM1_ESM.pdf]

Facilitators and Barriers in Obtaining Informed Consent for Neonatal Research: A Scoping Review

Supplementary Materials

Kelly K. Storm[1*], Veroni Stolk[1], Wes Onland[2,3], Sylvia A. Obermann-Borst[4], Anne Smits[5,6], Irwin K.M. Reiss[1], Anton H. van Kaam[2,3], Sinno H.P. Simons[1], G. Jeroen Hutten[2,3]

Affiliations

1. Department of Neonatal and Pediatric Intensive Care, Division of Neonatology, Erasmus University Medical Center – Sophia Children's Hospital, Rotterdam, The Netherlands
2. Department of Neonatology, Emma Children's Hospital, Amsterdam UMC, Amsterdam, The Netherlands
3. Amsterdam Reproduction & Development Research Institute, Amsterdam, The Netherlands
4. Care4Neo, Neonatal Patient and Parent Advocacy Organization, Rotterdam, The Netherlands
5. Neonatal Intensive Care Unit, University Hospitals Leuven, Leuven, Belgium
6. Department of Development and Regeneration, KU Leuven, Leuven, Belgium

* Corresponding author

k.storm@erasmusmc.nl

Online Resource 1. Overview of search databases and search strings

**Database**	**Search**
Medline	(exp *"Informed Consent"/ OR (consent* OR ((research* OR trial*) ADJ6 (participat* OR recruitment*))).ti.) **AND** (exp "Intensive Care Units, Neonatal"/ OR "Intensive Care, Neonatal"/ OR exp "Infant"/ OR (((neonatal*) ADJ3 (intensive* OR critical*) ADJ3 (care*)) OR NICU OR neonat* OR preterm* OR premature* OR pre-term* OR pre-mature* OR infant* OR baby OR babies OR newborn* OR new-born* OR newly-born*).ab,ti,kf.) **NOT** (news OR congres* OR abstract* OR book* OR chapter* OR dissertation abstract*).pt.
Embase	('informed consent'/exp/mj OR 'parental consent'/exp/mj OR (consent* OR ((research* OR trial*) NEAR/6 (participat* OR recruitment*))):ti) **AND** ('neonatal intensive care unit'/exp OR 'newborn intensive care'/exp OR 'infant'/exp OR (((neonatal*) NEAR/3 (intensive* OR critical*) NEAR/3 (care*)) OR NICU OR neonat* OR preterm* OR premature* OR pre-term* OR pre-mature* OR infant* OR baby OR babies OR newborn* OR new-born* OR newly-born*):ab,ti,kw) **NOT** ([Conference Abstract]/lim OR [preprint]/lim)
Web of Science	TI=(consent* OR ((research* OR trial*) NEAR/5 (participat* OR recruitment*))) **AND** TS=(((neonatal*) NEAR/2 (intensive* OR critical*) NEAR/2 (care*)) OR NICU OR neonat* OR preterm* OR premature* OR pre-term* OR pre-mature* OR infant* OR baby OR babies OR newborn* OR new-born* OR newly-born*) **NOT** DT=(Meeting Abstract OR Meeting Summary)
Cochrane CENTRAL	((consent* OR ((research* OR trial*) NEAR/6 (participat* OR recruitment*))):ti) **AND** ((((neonatal*) NEAR/3 (intensive* OR critical*) NEAR/3 (care*)) OR NICU OR neonat* OR preterm* OR premature* OR pre-term* OR pre-mature* OR infant* OR baby OR babies OR newborn* OR new-born* OR newly-born*):ab,ti,kw) **NOT** ("conference abstract":kw)
CINAHL	(MM "Consent" OR TI(consent* OR ((research* OR trial*) N6 (participat* OR recruitment*)))) **AND** (MH "Intensive Care Units, Neonatal" OR MH "Intensive Care, Neonatal+" OR MH "Infant+" OR TI(((neonatal*) N3 (intensive* OR critical*) N3 (care*)) OR NICU OR neonat* OR preterm* OR premature* OR pre-term* OR pre-mature* OR infant* OR baby OR babies OR newborn* OR new-born* OR newly-born*) OR AB(((neonatal*) N3 (intensive* OR critical*) N3 (care*)) OR NICU OR neonat* OR preterm* OR premature* OR pre-term* OR pre-mature* OR infant* OR baby OR babies OR newborn* OR new-born* OR newly-born*))
Google Scholar	'parental|informed consent' trial|trials|research|study<br>NICU|neonate|neonates|neonatal|newborn|infant|infants|preterm|premature

Online Resource 2. Facilitators of parental informed consent in neonatal research

Influencer	Significant facilitator	Facilitator	Not of influence
Patient characteristics			
Higher (perceived) severity of illness	Bauer 2021; Hulst 2005; Weiss & Olszewski 2021		Weiss & Guttmann 2021; Weiss & Olszewski 2021; Zupancic 1997
Younger postnatal age	Bauer 2021		Hulst 2005; Weiss & Guttmann 2021; Weiss & Olszewski 2021
Longer duration of NICU stay	Hulst 2005		
Parent/caregivers			
**Characteristics**			
Caucasian ethnicity	Israel 2025; Weiss & Olszewski 2021		Bauer 2021; Shah 2017; Weiss & Guttmann 2021
High income (> $55 000 annually)	Weiss & Olszewski 2021		Shah 2017
**Attitudes**			
Altruism	Bauer 2021; Shah 2017; Weiss & Guttmann 2021; Zupancic 1997	Ballard 2004; Cartwright 2011; Dahan 2020; Mason 2000; Nordheim 2018; O'Shea 2018; Sloss 2021; Van der Vaart 2024; Weiss 2024; Zupancic 1997	
Contribute to knowledge and science	Bauer 2021; Shah 2017; Weiss & Guttmann 2021	Dahan 2020; Nordheim 2018; Van der Vaart 2024; Weiss 2024	

Belief in the specific study	Weiss & Guttmann 2021	Ballard 2002; Sawyer 2017	
Positive attitude towards research in general	Zupancic 1997	Nordheim 2018; Sawyer 2017	
Fear for regret not joining	Weiss & Guttmann 2021		
Positive experience with previous research		Weiss 2024	
Desire to "give back"		Weiss 2024	
Relational			
Trust in researcher or in person who seeks consent	Shah 2018; Weiss & Guttmann 2021; Weiss & Olszewski 2021	Nordheim 2018	
Trust in medical team		Dahan 2020	Weiss & Olszewski 2021
Positive attitude towards researcher	Weiss & Guttmann 2021		
Perceived freedom to make consent decision	Zupancic 1997		
Desire to be a good patient		Dahan 2020	
Feeling pressured or obliged		Dahan 2020	
To help researcher		Sloss 2021	
Informed consent process related			
Sufficient time to make a decision		Cartwright 2011; Hoehn 2009	

Reading consent form carefully	Weiss & Guttmann 2021		
Discussion with others before deciding	Weiss & Guttmann 2021		
Positive first impression	Weiss & Guttmann 2021		
Sufficient information to make a decision		Nordheim 2018	
Concerns and questions answered by clinician		Cartwright 2011	
Clear explanation of the study		Ballard 2004	
Deferred consent		Sloss 2021	
Study characteristics			
Benefit to child	Bauer 2021; Shah 2017; Weiss & Guttmann 2021; Zupancic 1997	Ballard 2004; Cartwright 2011; Dahan 2020; Mason 2000; Nordheim 2018; O'Shea 2018; Sawyer 2017; Sloss 2021; Van der Vaart 2024; Weiss 2024	
Minimal risk or burden to child or parent	Zupancic 1997	Ballard 2004; Cartwright 2011; Mason 2000; Nordheim 2018; Sawyer 2017; Sloss 2021; Van der Vaart 2024; Weiss 2024	Bauer 2021
Belief in intervention	Bauer 2021	Sawyer 2017	
Chance on the treatment	Weiss & Guttmann 2021		
Active comparator	Hanvey 2019		
Short study length	Hanvey 2019		

Simplicity of the study design	Sawyer 2017
Benefit to parent	Sawyer 2017

Online Resource 3. Barriers to parental informed consent in neonatal research

Influencer	Significant barrier	Barrier	Not of influence
Patient characteristics			
Lower (perceived) severity of illness	Bauer 2021; Hulst 2005; Weiss & Olszewski 2021		Weiss & Guttmann 2021; Weiss & Olszewski 2021; Zupancic 1997
Higher (perceived) severity of illness		O'Shea 2018	Weiss & Guttmann 2021; Weiss & Olszewski 2021; Zupancic 1997
Older postnatal age	Bauer 2021		Hulst 2005; Weiss & Guttmann 2021; Weiss & Olszewski 2021
Shorter duration of NICU stay	Hulst 2005		
Parent/caregivers			
**Characteristics**			
Black ethnicity	Israel 2025; Weiss & Olszewski 2021		Bauer 2021; Shah 2017; Weiss & Guttmann 2021
Public health insurance	Weiss & Olszewski 2021		
**Attitudes**			
Parental fear or distress	Weiss & Guttmann 2021	Cakici 2023; Dahan 2020; Mason 2000; Sloss 2021	

Negative attitude towards research in general	Zupancic 1997	Mason 2000; Sloss 2021	
Decisional conflict	Shah 2017; Weiss & Guttmann 2021		
Guinea pig concerns	Weiss & Guttmann 2021		Shah 2017
Lack of belief in specific study		Cakici 2023; Sloss 2021	
Fear for loss of control	Weiss & Guttmann 2021	Weiss 2024	
Risk for discrimination		Cakici 2023; Weiss 2024	
Fear for regret joining	Weiss & Guttmann 2021		
Preference for clinical team member to obtain consent	Weiss & Guttmann 2021		
Preference for standard care		Weiss 2024	
Privacy concerns		Weiss 2024	
Relational			
Inappropriate interaction by researcher or clinician		Dahan 2020; Mason 2000; Weiss 2024	
Lack of trust in researcher	Shah 2017	Mason 2000	
Feeling pressured or obliged	Zupancic 1997		
Informed consent process related			

Poor comprehension of the study information		Dahan 2020	Shah 2017; Weiss & Olszewski
Insufficient time to make the decision	Weiss & Guttmann 2021	Hoehn 2009	
Complex consent process	Zupancic 1997		Bauer 2021
Absence of one parent		Dahan 2020; Hoehn 2009	
Too many proposals for other studies		Dahan 2020; Weiss 2024	
Negative first impression	Weiss & Guttmann 2021		
Study characteristics			
Risk to child	Zupancic 1997	Mason 2000; Nordheim 2018; O'Shea 2018; Weiss 2024	Bauer 2021; Shah 2017
Logistics or burden of participation	Weiss & Guttmann 2021	Hoehn 2009; Mason 2000; Nordheim 2018; Weiss 2024	Bauer 2021
Lack of direct benefit to child	Zupancic 1997	Weiss 2024	
Placebo	Hanvey 2019	Weiss 2024	
Randomization concerns		Weiss 2024	Hanvey 2019
Study intervention			
Blood sampling		Cakici 2023	Hanvey 2019
Future use of data		Cakici 2023	

Potential loss of confidentiality		Cakici 2023
Genetic nature		Cakici 2023
Long study length	Hanvey 2019	
Blinding		Weiss 2024

Online Resource 4. Factors not influencing parental informed consent in neonatal research

		Factors not of influence	References
Patient characteristics		Gestational age	Bauer 2021; Hanvey 2019; Hulst 2005; Shah 2017; Weiss & Guttmann 2021; Weiss & Olszewski 2021
		Birth weight	Bauer 2021; Hulst 2005; Weiss & Olszewski 2021
		Sex	Bauer 2021; Hulst 2005
		Postmenstrual age	Bauer 2021
		Singleton	Bauer 2021
		Underlying disease	Hulst 2005
		Inborn	Weiss & Olszewski 2021
		Death during admission	Hulst 2005
Parents / caregivers	*Characteristics*	Parental age	Bauer 2021; Weiss & Guttmann 2021; Weiss & Olszewski 2021
		Parental education	Bauer 2021; Shah 2017; Weiss & Olszewski 2021
		Language	Shah 2017; Weiss & Olszewski 2021
		Religious (yes/no)	Bauer 2021
		Employment status	Bauer 2021
		Single parent family	Bauer 2021
		Other children with chronic health problems	Weiss & Olszewski 2021
		Other children had been in NICU	Weiss & Olszewski 2021
	Attitudes	Previous parental research involvement	Bauer 2021; Shah 2017; Weiss & Olszewski 2021; Zupancic 1997
		Fear that not participating will arouse negativity	Bauer 2021
		Perceived impact of declining on infant's care	Sloss 2021
		Reliance on phycisian's advice	Zupancic 1997
Informed consent process		Role of individual approaching	Bauer 2021; Shah 2017; Weiss & Guttmann 2021
		Mother or father respondent	Weiss & Olszewski 2021; Shah 2017
		Timing of approach (pre- vs post-natal)	Shah 2017
		Consent over phone	Weiss & Guttmann 2021
Study characteristics		Drug administration route	Hanvey 2019
		Drug dosing	Hanvey 2019
		Treatment length	Hanvey 2019
		Funding source	Hanvey 2019
		Study phase	Hanvey 2019
		Extra attention by participating	Bauer 2021
